# Spectrum and prevalence of *BRCA1/2* germline mutations in Pakistani breast cancer patients: results from a large comprehensive study

**DOI:** 10.1186/s13053-019-0125-5

**Published:** 2019-09-11

**Authors:** Muhammad Usman Rashid, Noor Muhammad, Humaira Naeemi, Faiz Ali Khan, Mariam Hassan, Saima Faisal, Sidra Gull, Asim Amin, Asif Loya, Ute Hamann

**Affiliations:** 1 Basic Sciences Research, Shaukat Khanum Memorial Cancer Hospital and Research Centre (SKMCH&RC), Lahore, Pakistan; 2 Molecular Genetics of Breast Cancer, German Cancer Research Center (DKFZ), Im Neuenheimer Feld 580, 69120 Heidelberg, Germany; 3 Clinical Research Office, SKMCH&RC, Lahore, Pakistan; 4 Leibniz Institute on Aging - Fritz Lipmann Institute, Jena, Germany; 5 Levine Cancer Institute, Charlotte, USA; 6 Pathology Department, SKMCH&RC, Lahore, Pakistan

**Keywords:** *BRCA1/2*, germline mutations, breast cancer, Pakistan

## Abstract

**Background:**

Pathogenic germline mutations in *BRCA1* and *BRCA2* (*BRCA1/2*) account for the majority of hereditary breast and/or ovarian cancers worldwide. To refine the spectrum of *BRCA1/2* mutations and to accurately estimate the prevalence of mutation in the Pakistani population, we studied 539 breast cancer patients selected for family history and age of diagnosis.

**Methods:**

Comprehensive screening for *BRCA1/2* germline mutations was performed using state-of-the-art technologies.

**Results:**

A total of 133 deleterious mutations were identified in 539 families (24.7%), comprising 110 in *BRCA1* and 23 in *BRCA2*. The prevalence of *BRCA1/2* small-range mutations and large genomic rearrangements was 55.4% (36/65) for families with breast and ovarian cancer, 27.4% (67/244) for families with two or more cases of breast cancer, 18.5% (5/27) for families with male breast cancer, and 12.3% (25/203) for families with a single case of early-onset breast cancer. Nine mutations were specific to the Pakistani population. Eighteen mutations in *BRCA1* and three in *BRCA2* were recurrent and accounted for 68.2% (75/110) and 34.8% (8/23) of all identified mutations in *BRCA1* and *BRCA2*, respectively. Most of these mutations were exclusive to a specific ethnic group and may result from founder effects.

**Conclusions:**

Our findings show that *BRCA1/2* mutations account for one in four cases of hereditary breast/ovarian cancer, one in five cases of male breast cancer, and one in eight cases of early-onset breast cancer in Pakistan. Our study suggests genetic testing of an extended panel of 21 recurrent *BRCA1/2* mutations for appropriately selected patients and their families in Pakistan.

## Background

Individuals harboring *BRCA1/2* germline mutations have high lifetime risks of breast and ovarian cancer. The identification of individuals harboring *BRCA1/2* mutations is crucial to assess their cancer risk, consider preventive measures and tailor cancer management strategies.

Several studies have investigated the prevalence of *BRCA1/2* small-range mutations and/or large genomic rearrangements (LGRs) with frequencies varying from 17.6% to 29.8% in white populations from Europe and Australia [1–5] and 9.4% to 21.7% in non-whites from Asia [6–8]. The prevalence and distribution of *BRCA1/2* mutations vary across populations, mainly due to population-specific recurrent or founder mutations. Accurate identification of the population-specific mutation spectrum is therefore the first step towards incorporating appropriate genetic *BRCA1/2* testing into clinical practice in a particular population. This information is not fully elucidated in Pakistan, a country with one of the highest rates of breast cancer in Asia.

To date, no large comprehensive studies evaluating the *BRCA1/2* mutations have been reported in the Pakistani population and mutations in males have not been identified so far. Small-range mutations were previously reported in 341 unselected breast and 120 ovarian cancer patients, in which the analysis was restricted to a few exons only [9]. We conducted two studies in early-onset and familial breast/ovarian cancer patients from Pakistan. In the initial study the complete coding regions and exon-intron boundaries of *BRCA1/2* were screened for small-range mutations in 176 patients [10]. In the other study 120 *BRCA1/2* small-range mutations negative patients were screened for LGRs [11]. Other Asian studies also had small sample sizes [12, 13], reported small-range mutations only [14, 15], and/or restricted LGR analyses to a small number of study participants [6, 16, 17].

Here, we refined the spectrum of *BRCA1/2* mutations and more precisely estimated the mutation frequencies including small-range mutations and LGRs in 539 early-onset and familial breast cancer patients from Pakistan.

## Methods

### Enrollment of families

**Fig. 1:**
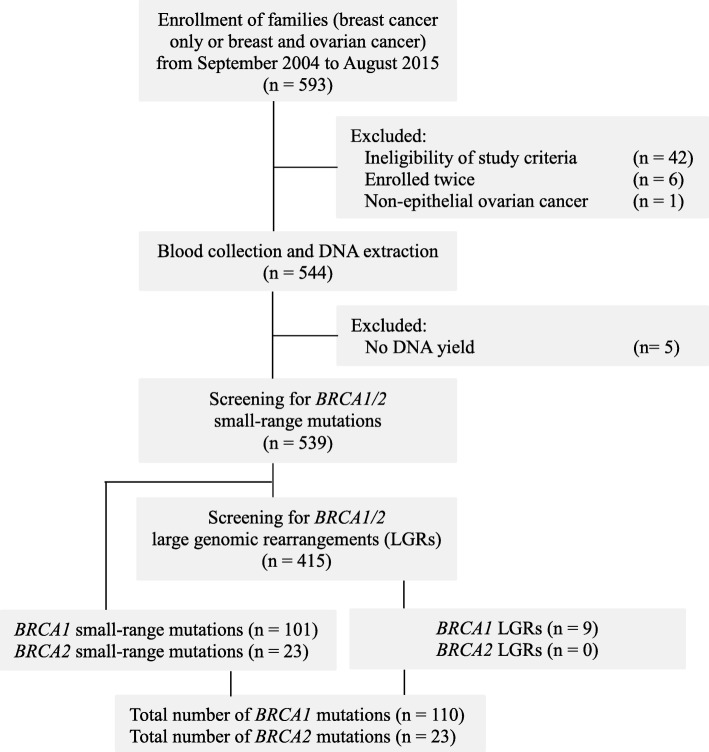
Flow diagram for the study participant’s enrollment, screening methods used and *BRCA1/2* mutations detected

Five hundred and ninety-three breast cancer only or breast and ovarian cancer families were enrolled through index breast and/or ovarian cancer patients who presented at the Shaukat Khanum Memorial Cancer Hospital and Research Centre (SKMCH&RC) in Lahore, Pakistan, from September 2004 to August 2015. The recruited families were classified into five risk groups based on family history of breast/ovarian cancer or age at diagnosis (Table 1) as described previously [19]. After enrollment, 54 families were excluded (Fig. 1), leaving 539 families in the study.

**Table 1:** *BRCA1/2* mutation frequencies according to family structure

Risk group	Phenotype of families	No. of families	No. of families with mutations (%) in						
			*BRCA1*			*BRCA2*			*BRCA1/2*
			Small-range	LGRs	All	Small-range	LGRs	All	
	All families	539	101 (18.7)	9 (1.7)	110 (20.4)	23 (4.3)	0 (0)	23 (4.3)	133 (24.7)^a^
	Female breast cancer families	447	67 (15.0)	7 (1.6)	74 (16.6)	18 (4.0)	0 (0)	18 (4.0)	92 (20.6)
A1	1 case ≤ 30 years	203	20 (9.8)	2 (1.0)	22 (10.8)	3 (1.5)	0 (0)	3 (1.5)	25 (12.3)
A2	2 cases, >1 diagnosed ≤50 years	131	20 (15.3)	4 (3.0)	24 (18.3)	6 (4.6)	0 (0)	6 (4.6)	30 (22.9)
A3	>3 cases, >1 diagnosed ≤50 years	113	27 (23.9)	1 (0.9)	28 (24.8)	9 (8.0)	0 (0)	9 (8.0)	37 (32.7)
A4	Male breast cancer families								
	>1 case of male breast cancer	27	1 (3.7)	0 (0)	1 (3.7)	4 (14.8)	0 (0)	4 (14.8)	5 (18.5)
B	Breast-ovarian cancer families								
	>1 breast cancer and >1 ovarian cancer	65	33 (50.8)	2 (3.0)	35 (53.8)	1 (1.5)	0 (0)	1 (1.5)	36 (55.4)

*LGRs* large genomic rearrangements

^a^Including 57 previously reported families [11, 18]

Clinical and histopathological data and comprehensive information on personal and family history of cancer(s), and ethnicity were obtained from all study participants. The Institutional Review Board of the SKMCH&RC approved the study. All study participants signed an informed written consent before providing a blood sample.

### *BRCA1/2* mutation screening

Genomic DNA was extracted from 9 to 18 ml of whole blood samples, as described previously [20]. The entire coding regions of the *BRCA1* (Genbank accession number U14680) and *BRCA2* (Genbank accession number U43746) genes including exon-intron boundaries were screened in 139 patient DNA samples for small-range mutations using denaturing high-performance liquid chromatography (DHPLC) analysis as previously described [21, 22]. Each sample revealing variants was sequenced as described elsewhere [18]. Four-hundred female patients selected based on triple-negative vs. non triple-negative breast cancer phenotype had been screened for small-range *BRCA1/2* mutations and 33 mutations were described [18]. All small-range mutation-negative patients had been screened for LGRs using multiplex ligation-dependent probe amplification and three LGRs were described [11]. For the current study, families were selected on the basis of family history of breast/ovarian cancer, male breast cancer or age at diagnosis.

### Mutation classification

All *BRCA1/2* alterations identified in the current study were classified into pathogenic mutations, variants of unknown significance, or polymorphisms. Pathogenic mutations were defined as (i) small-range mutations which affect one or a few nucleotides including frameshift, nonsense, or splice-site mutations and generate a premature termination codon, except *BRCA2* exon 27 variants generating a premature termination codon after codon 3010 [23] and (ii) LGRs that span one or more exons. Mutations were designated using the Human Genome Variation Society (HGVS) and the Breast Cancer Information Core (BIC) committee nomenclature.

All identified mutations were searched in various mutation databases including BIC (https://research.nhgri.nih.gov/bic/), ClinVar (http://www.ncbi.nim.nih.gov/clinvar/), LOVD (http://databases.lovd.nl/shared/genes/BRCA2), ARUP (http://arup.utah.edu/database/BRCA/), and BRCA Exchange (http://brcaexchange.org/). Mutations not reported in these databases were considered as novel and specific to Pakistani population.

### Statistical analyses

Distribution of clinical and histopathological characteristics between *BRCA1/2* carriers and non-carriers were estimated using Fisher’s exact test for categorical variables and the Wilcoxon rank-sum test for quantitative variables. All statistical tests were two-sided. Results were considered significant at a *p* value of <0.05. All statistical analyses were done using StatXact 4 for Windows (Cytel, Cambridge, USA) and R, version 2.1.

## Results

A total of 539 index patients from unrelated families were enrolled and stratified into five risk groups (Table 1). The mean age of disease onset was 35.4 years (range 18-78) for female breast cancer (n=502), 45.4 years (range 23-66) for ovarian cancer (n=30) and 54.5 years (range 27-76) for male breast cancer (n=27) patients.

### Spectrum of *BRCA1/2* mutations

Evaluation of pooled data from 539 patients yielded 71 distinct pathogenic mutations in 133 families (24.7%) (Table 1). Fifty-three *BRCA1* mutations were detected in 110 families (20.4%) and 18 *BRCA2* mutations in 23 families (4.3%). Five mutations in *BRCA1* (9.4%) and four mutations in *BRCA2* (22.2%) were novel (Table 2). The phenotypes of all families carrying *BRCA1/2* mutations are presented in Table 3.

**Table 2:** Deleterious *BRCA1/2* germline mutations in Pakistani breast/ovarian cancer families

Family	Exon	BIC designation			HGVS designation		Mutation type^b^	Reported in databases (No. of entries)^c^
		Nucleotide	Codon	Designation	Nucleotide change^a^	Effect on protein		
*BRCA1*-small-range mutations								
432	2	185	23	185insA	c.66dup	p.(Glu23Argfs*18)	FS	BIC (32)^d^
723	2	185	23	185delAG	c.68_69del	p.(Glu23Valfs*17)	FS	BIC (2036)^d^
372	Intron 4	IVS4-2	-	IVS4-2A>G	c.135-2A>G	Splice site	SP	BIC (1)
254^e^	7	454	112	454delA	c.335del	p.(Asn112Ilefs*7)	FS	ClinVar (2)
449	7	509	130	Y130X	c.390C>G	p.(Tyr130*)	NS	LOVD (3)
296, 317, 340^e^, 511, 521, 626^e^, 747	11	804	229	804delT	c.685del	p.(Ser229Leufs*5)	FS	BIC (2)
470^e^	11	903	262	Q262X	c.784C>T	p.(Gln262*)	NS	ClinVar (3)
711	11	1014	299	1014delGT	c.895_896del	p.(Val299Argfs*4)	FS	BIC (2)
669^e^	11	1127	336	1127delA	c.1008del	p.(Glu337Lysfs*4)	FS	No^d^
748	11	1307	396	1307delT	c.1188del	p.(Asp396Glufs*14)	FS	LOVD (1)
241^e^	11	1309	397	1309delA	c.1190del	p.(Asp397Alafs*13)	FS	ClinVar (3)
722	11	1518	485	1518_1572dup55	c.1399_1453dup	p.(Ala485Glufs*13)	FS	No
336^e^	11	1590	491	Q491X	c.1471C>T	p.(Gln491*)	NS	BIC (4)^d^
N12	11	1898	593	1898delTATGGAA	c.1779_1785del	p.(Met594Serfs*3)	FS	LOVD (2)
N28, 328^e^, 557^e^	11	1912	598	L598X	c.1793T>G	p.(Leu598*)	NS	BIC (1)^d^
574^e^	11	2080	655	2080insA	c.1961dup	p.(Tyr655Valfs*18)	FS	BIC (13)^d^
488^e^	11	2268	717	E717X	c.2149G>T	p.(Glu717*)	NS	ClinVar (2)
236^e^, 283^e^, 489^e^, 493^e^, 593	11	2388	757	2388delG	c.2269del	p.(Val757Phefs*8)	FS	BIC (10)^d^
363	11	2433	772	2433delGT	c.2314_2315del	p.(Val772Thrfs*4)	FS	LOVD (2)
550^h^	11	2457	780	Q780X	c.2338C>T	p.(Gln780*)	NS	BIC (36)
362, 469	11	2459	780	2459delGGAA	c.2340_2343del	p.(Glu781Valfs*10)	FS	LOVD (2)
421^e^, 442, 510^e^, 542, 619^e^	11	2524	802	2524delTG	c.2405_2406del	p.(Val802Glufs*7)	FS	BIC (5)^d^
N34	11	2657	846	2657delAAT-insG	c.2538_2540delinsG	p.(Met847Glyfs*4)	FS	LOVD (2)
415^e^, 660^e^	11	2722	868	S868X	c.2603C>G	p.(Ser868*)	NS	BIC (11)^d^
411^e^	11	3090	991	K991X	c.2971A>T	p.(Lys991*)	NS	ClinVar (2)
247^e^	11	3100	994	3100delGT	c.2981_2982del	p.(Cys994*)	FS	ClinVar (5)
299	11	3248	1043	3248delTATTAATGAA	c.3129_3138del	p.(Asn1043Lysfs*2)	FS	ClinVar (3)
N13^e^, 399^e^	11	3458	1113	3458delTGA	c.3339_3341del	p.(Tyr1113*)	FS	ClinVar (2)
610	11	3531	1138	G1138X	c.3412G>T	p.(Gly1138*)	NS	ClinVar (3)
N25	11	3596	1159	3596delAAAG	c.3477_3480del	p.(Ile1159Metfs*50)	FS	BIC (3)
279^e^, 445^e^	11	3717	1200	Q1200X	c.3598C>T	p.(Gln1200*)	NS	BIC (21)^d^
382	11	3726	1203	R1203X	c.3607C>T	p.(Arg1203*)	NS	BIC (36)
646	11	3819	1234	3819delGTAAA	c.3700_3704del	p.(Val1234Glnfs*8)	FS	BIC (61)
210^e^, 211^e^, 313^e^, 316, 332^e^, 463, 520, 624, 652^e^, 653	11	3889	1257	3889delAG	c.3770_3771del	p.(Glu1257Glyfs*9)	FS	BIC (23)^d^
N4^e^, 687, 724, 743	11	4184	1355	4184delTCAA	c.4065_4068del	p.(Asn1355Lysfs*10)	FS	BIC (144)^d^
318^e^	12	4302	1395	Q1395X	c.4183C>T	p.(Gln1395*)	NS	BIC (28)^d^
408^e^	13	4446	1443	R1443X	c.4327C>T	p.(Arg1443*)	NS	BIC (128)
523^e^, 555, N18, 598^e^, 612, 621	Intron 14	IVS14-1	-	IVS14-1G>A	c.4485-1G>A	Splice site	SP	BIC (2)^d^
220^e^, 275^e^, 512^e^	15	4627	1503	S1503X	c.4508C>A	p.(Ser1503*)	NS	BIC (1)^d^
609^e^	15	4784	1558	4784delG	c.4665del	p.(Arg1555Serfs*4)	FS	No
611^e^	16	4981	1621	4981delA	c.4862del	p.(Asp1621Valfs*12)	FS	No
249^e^, 658	17	5154	1679	5154delC	c.5035del	p.(Leu1679*)	FS	BIC (2)
276^e^, 679	Intron 17	IVS17+1	-	IVS17+1G>A	c.5074+1G>A	Splice site	SP	BIC (3)
685	20	5358	1747	5358delC	c.5239del	p.(Gln1747Lysfs*18)	FS	LOVD (2)
734	20	5385	1756	5385dupC	c.5266dup	p.(Gln1756Profs*74)	FS	LOVD (376)
706	Intron 20	IVS20-1	-	IVS20-1G>C	c.5278-1G>C	Splice site	SP	LOVD (5)^d^
678	21	5429	1771	5429dupG	c.5310dup	p.(Pro1771Alafs*59)	FS	LOVD (1)
278, 338^e^	22	5480	1787	5480delTG	c.5361_5362del	p.(Cys1787Trpfs*42)	FS	ClinVar (3)
682	22	5496	1793	K1793X	c.5377A>T	p.(Lys1793*)	NS	ClinVar (1)
248^e^	Intron 23	IVS23-2	-	IVS23-2A>T	c.5468-2A>T	Splice site	SP	ClinVar (1)
260, 264, 329^e^, 377^e^, 389, 439, 481, 501, 522	24	5622	1835	R1835X	c.5503C>T	p.(Arg1835*)	NS	BIC (74)^d^
*BRCA1*-large genomic rearrangements^e^								
229, 291, 314, 379, 406, 498, 549	1-2	-	-	del exon 1-2	g.41271967_41308900del^f^		LGR	(42)^g^
261, 719	21-24	-	-	del exon 21-24	g.41172653_41205744del^f^		LGR	No
*BRCA2*-small-range mutations								
497, 700	3	320	31	W31X	c.92G>A	p.(Trp31*)	NS	ClinVar (4)
N26	Intron 4	IVS4-2	-	IVS4-2A>G	c.426-2A>G	Splice site	SP	ClinVar (4)
545	9	993	255	993delCACAA	c.765_769del	p.(Asn255Lysfs*19)	FS	No
330	10	1528	434	1528delAAAA	c.1300_1303del	p.(Lys434Glufs*25)	FS	ClinVar (2)
602	11	3048	941	3048delA	c.2820del	p.(Val941Cysfs*19)	FS	No
206	11	3063	945	3063delA	c.2835del	p.(Asp946Ilefs*14)	FS	ClinVar (2)
505	11	4088	1287	4088delA	c.3860del	p.(Asn1287Ilefs*6)	FS	BIC (2)
222, 407^h^, 525, 540^h^	11	5450	1741	5450delGTAA	c.5222_5225del	p.(Ser1741Thrfs*35)	FS	BIC (1)
627, 684	11	5910	1894	Y1894X	c.5682C>A	p.(Tyr1894*)	NS	BIC (3)
295^e^	11	5950	1908	5950delCT	c.5722_5723del	p.(Leu1908Argfs*2)	FS	BIC (43)^d^
447	11	6696	2156	6696delTC	c.6468_6469del	p.(Gln2157Ilefs*18)	FS	BIC (24)^d^
548^h^	11	7044	2274	7044delAAGAG	c.6816_6820del	p.(Gly2274Alafs*17)	FS	ClinVar (6)
579	15	7803	2526	7803delA	c.7575del	p.(Ala2526Glnfs*2)	FS	LOVD (2)
492	Intron 17	IVS17+2	-	IVS17+2C>A	c.7976+2C>A	Splice site	SP	ClinVar (1)
713	20	8773	2849	8773delAA	c.8545_8546del	p.(Lys2849Glyfs*19)	FS	No
702	20	8779	2860	8779_8798dup20	c.8551_8570dup	p.(Lys2860Asnfs*10)	FS	No
207^h^	21	8897	2890	8897insT	c.8669dup	p.(Thr2891Asnfs*16)	FS	ClinVar (1)
538	Intron 21	IVS21+4	-	IVS21+4A>G	c.8754+4A>G	Splice site	SP	BIC (7)

^a^Numbering starts at the first A of the first coding ATG (located in exon 2) of NCBI GenBank accession number U14680 (*BRCA1*) and U43746 (*BRCA2*)

^b^*FS* frameshift mutation, *LGR* large genomic rearrangement, *MS* missense mutation, *NS* nonsense mutation, *SP* splice-site mutation

^c^*BIC* Breast Cancer Information Core database (https://research.nhgri.nih.gov/projects/bic/), *LOVD* Leiden Open Variation Database (http://databases.lovd.nl/shared/genes/BRCA2); ClinVar (https://www.ncbi.nlm.nih.gov/clinvar/), date last accessed June 26, 2018

^d^Previously reported in Pakistani breast/ovarian cancer cases [9, 10]

^e^Families and mutation description have been previously reported [11, 18]

^f^Genomic locale for chromosome 17, from the UCSC genome browser, Feb 2009 assembly

^g^Not available in databases; reported in various studies [1, 11]

^h^Families with male breast cancer

**Table 3:** Characteristics of the 133 families with deleterious *BRCA1/2* mutations

Family	No. of cancers		Age at onset (years)		Other cancer(s)^c^ (age at onset in years)	Ethnicity
	Female BC (Bilateral)	OC (OC+BC)	BC	OC		
Families carrying *BRCA1-*small-range mutations						
236^a^	1	-	22^b^	-	-	Pathan
316	1	-	25^b^	-	-	Punjabi
264	1	-	26^b^	-	-	Punjabi
706	1	-	26^b^	-	Uterus (67)	Punjabi
N12	1	-	26^b^	-	-	Punjabi
624	1	-	27^b^	-	-	Punjabi
N25	1	-	28^b^	-	-	Punjabi
276^a^	1	-	28^b^	-	-	Punjabi
610	1	-	28^b^	-	-	Punjabi
678	1	-	28^b^	-	-	Punjabi
411^a^	1	-	29^b^	-	Stomach (70)	Punjabi
724	1	-	29^b^	-	Renal (48), lung (65), throat (65), unknown	Punjabi
N28	1	-	30^b^	-	-	Punjabi
279^a^	1(1)	-	27/36^b^	-	-	Punjabi
278	2	-	25^b^,32	-	-	Kashmiri
332^a^	2	-	26^b^,51	-	Leukemia (45)	Punjabi
682	2	-	28^b^,40	-	Uterus (<62,65), throat (<72)	Punjabi
N18	2	-	29^b^,<50	-	-	Punjabi
421^a^	2	-	30^b^,33	-	-	Punjabi
432	2	-	30^b^,53	-	Skin (12), oral (54)	Punjabi
520	2	-	30^b^,47	-	Uterus (32)	Punjabi
449	2	-	32^b^,55	-	-	Punjabi
557^a^	2	-	32^b^,45	-	Unknown (<55), renal (70)	Punjabi
747	2	-	33^b^,38	-	-	Unknown
722	2	-	20,34^b^	-	Unknown (<18,<40)	Punjabi
687	2	-	37^b^,45	-	-	Punjabi
470^a^	2	-	40^b^,40	-	Stomach (46), colon (59), lung	Punjabi
510^a^	2	-	40^b^,55	-	-	Punjabi
N13^a^	2	-	40^b^,>50	-	-	Punjabi
593	2	-	43,44^b^	-	Leukemia (22)	Pathan
299	2(1)	-	24/27^b^,55	-	-	Punjabi
660^a^	2(1)	-	25/26^b^,70	-	Bladder	Punjabi
260	2(1)	-	25/26^b^,28	-	-	Punjabi
511	2(1)	-	30/33^b^,<32	-	Brain (75)	Punjabi
N34	3	-	24^b^,<30,31	-	-	Punjabi
669^a^	3	-	25^b^,<40,<50	-	Brain (<78), oral (<80)	Punjabi
685	3	-	26^b^,26,?	-	Blood (2x)	Mohajir
723	3	-	28^b^,40,?	-	-	Pathan
612	3	-	29^b^,<30,40	-	Throat (45), uterus (48)	Punjabi
313^a^	3	-	30^b^, 48,?	-	-	Punjabi
336^a^	3	-	23,30^b^,38	-	Prostate (29)	Punjabi
493^a^	3	-	35^b^,55,>55	-	-	Pathan
382	3	-	36^b^,50,?	-	-	Punjabi
489^a^	3	-	25,42^b^,45	-	-	Punjabi
743	3	-	40,44^b^,62	-	Bone (60), leukemia (60)	Pathan
658	3	-	26,44^b^,50	-	-	Punjabi
377^a^	3	-	31,50^b^,85	-	Thyroid (59), intestine (70), bladder (75), liver	Punjabi
550^d^	3	-	50^b^,55,>50	-	Lung, unknown	Punjabi
372	3(1)	-	21/21^b^,29,45	-	Squamous cell carcinoma scalp (22)^b^	Pathan
626^a^	3(1)	-	35^b^,36/37, 42	-	-	Balochi
389	3(1)	-	22/32,42,48^b^	-	Brain (36), uterus (70)	Punjabi
247^a^	4	-	27,28^b^,40,42	-	Uterus (31, 55)	Siriaki
652^a^	4	-	31^b^,33,42,50	-	-	Punjabi
362	4	-	31,32,35^b^,45	-	Liver (>40), abdomen	Pathan
399^a^	4	-	43, 44^b^,50,?	-	Abdomen (45), lung (45), prostate (53)	Punjabi
338^a^	4	-	30,40,44,48^b^	-	Stomach (73)	Punjabi
408^a^	4(1)	-	24^b^,31/31,33,50	-	Abdomen (54), esophagus (74)	Punjabi
521	4(1)	-	25/38^b^,27,33,70	-	Stomach (60, 65), lung, unknown	Punjabi
653	5	-	24,32,35^b^,37,50	-	Colon (42), throat (66)	Punjabi
734	5	-	37,38^b^,55,?,?	-	-	Punjabi
296	7(1)	-	21^b^,<30,34,43,44/44,51,52	-	-	Punjabi
439	8	-	30,<35,40^b^,40,40,40,>50,>65	-	Uterus (40), prostate, unknown	Punjabi
249^a^	8(1)	-	30/31^b^,37,45,?,?,?,?,?	-	-	Punjabi
619^a^	1	1	30^b^	>50	-	Punjabi
646	1	1(1)	34^b^	36^b^	-	Punjabi
748	1	1(1)	47^b^	52^b^	Skin (45), liver (50)	Punjabi
542	1	2(1)	40^b^	46^b^,70	Leukemia (74)	Punjabi
210^a^	1	4	45^b^	?,?,?,?	Brain (32), abdomen, lung, leukemia	Punjabi
241^a^	2	1	29^b^,64	35	Lymphoma	Punjabi
598^a^	2	1	30^b^,56	>50	Stomach (>50, >50), tongue (>50)	Punjabi
463	2	1	35^b^,58	48	-	Punjabi
481	2	1	<25,47^b^	36	Lung (55), uterus	Punjabi
621	2	1	50,>60	50^b^	Prostate (65)	Punjabi
211^a^	2	1(1)	26^b^,50	50	-	Punjabi
415^a^	2	1(1)	34,35^b^	35^b^	Leukemia (45), unknown	Punjabi
679	2	1(1)	28^b^,<33	49^b^	-	Punjabi
N4^a^	2	1 (1)	41^b^,45	45	Uterus (38)	Punjabi
488^a^	2	2(1)	40^b^,55	42^b^,45	-	Punjabi
555	2	2(1)	29^b^,36	36^b^,>50	Leukemia (10), vocal cord (45)	Punjabi
317	2	3(1)	41,46	47^b^,52,55	Fallopian tube (47)^b^	Punjabi
318^a^	2	4(2)	40,46^b^	40,42^b^,44,58	Bladder (50, 50)	Pathan
442	2(1)	2	34,50/50^b^	28,52	Leukemia (15)	Punjabi
283^a^	2(1)	3	34/38^b^,56	54,55,65	-	Pathan
254^a^	3	1	27,32^b^,43	41	Brain	Punjabi
711	3	1	40^b^,?,?	?	Gall bladder	Sindhi
445^a^	3	1(1)	44^b^,>60,73	74	Gall bladder	Punjabi
363	3	2	32,35,70	47^b^, ?	Lung (65), oral (70), liver	Kashmiri
329^a^	3	3	34^b^,39,?	39,<50,?	-	Punjabi
609^a^	4(1)	1	29^b^,31,48/55,65	30	-	Mohajir
328^a^	4	1	29,30^b^,31,39	55	-	Punjabi
501	4	1	34^b^, 35, >50,?	?	Brain (42)	Punjabi
522	4	2(1)	30^b^,<40,45,45	35,60	Uterus (41)	Punjabi
611^a^	4	2(1)	31^b^,36,37,42	50,55	Blood (30)	Punjabi
523^a^	3(1)	4	39/46,40,53^b^	30,45,51,60	-	Punjabi
275^a^	4(1)	1(1)	34/40^b^,40,42,50	40	-	Punjabi
469	5	1(1)	29^b^,29,<35,>35,>55	32^b^	-	Mohajir
340^a^	6	1	34^b^,42,<50,<50,<50,<50	54	-	Balochi
574^a^	6	2	32,32,35,45^b^,48,>50	48,55	-	Mohajir
512^a^	6(1)	1	<25,<30,<40,46/55^b^,<50,>50	<50	Uterus (<50)	Kashmiri
248^a^	7	2(2)	23^b^,<25,34,<40,<46,<60,?	23^b^,<60	-	Punjabi
220^a^	8	1(1)	25,27^b^,<30,53,58,63,77	<30	-	Punjabi
Families carrying *BRCA1-*LGRs^a^						
229	1	-	28^b^	-	-	Punjabi
379	2	-	29,31^b^	-	Liver (38)	Punjabi
261	2	-	33,34^b^	-	-	Punjabi
406	2	-	39^b^,40	-	Abdomen (65)	Punjabi
498	2	-	40,41^b^	-	-	Siriaki
549	2	-	38,72^b^	-	Unknown	Punjabi
314	6	-	32^b^,42,56,70,?,?	-	Uterus (54), pharynx (59), brain (63), abdomen	Punjabi
291	3	1	39,42,48	48^b^	Stomach, brain	Punjabi
719	3(1)	1	>40,42^b^,?	?	-	Punjabi
Families carrying *BRCA2-*small-range mutations						
330	1	-	29^b^	-	Lung (48, 58, 66), tongue (55)	Punjabi
206	1	-	30^b^	-	-	Pathan
540^d^	1	-	67^b^	-	-	Mohajir
207^d^	1	-	76^b^	-	Intestine (60)	Punjabi
295^a^	1(1)	-	23/23^b^	-	Leukemia (49), esophagus (50)	Punjabi
N26	2	-	26^b^,35	-	-	Pathan
602	2	-	31^b^,43	-	-	Punjabi
492	2	-	38^b^,39	-	-	Mohajir
505	2	-	43,46^b^	-	-	Pathan
713	2	-	35,56^b^	-	-	Kashmiri
700	2(1)	-	35/43^b^,46	-	Throat (72)	Punjabi
627	2(1)	-	42^b^,51/51	-	-	Pathan
545	3	-	35,36^b^,47	-	Brain (50), uterus (50), bone (54)	Punjabi
497	3	-	51^b^,55,50	-	Brain	Siriaki
548^d^	3	-	45,50,69^b^	-	-	Pathan
702	4	-	26,30^b^,<33,70	-	Throat (72)	Punjabi
579	4	-	35,49,<50,51^b^	-	Oral (35), gall bladder (42)	Kashmiri
407^d^	4	-	31,45(male),45,54^b^	-	Esophagus (39,59), leukemia (64)	Mohajir
538	5	-	34^b^,38,45,50,58	-	Retinoblastoma (3), pancreas (75), liver (83)	Pathan
684	5	-	40^b^,45,48,50,<57	-	Throat (<48, <82), stomach (53), intestine (60)	Pathan
447	3(1)	2	31^b^,<50,55/65	50,>50	Abdomen (>50), colon (62), brain (65)	Punjabi
525	4(1)	1	32,33/35^b^,<50,60	<45	Bladder (>60)	Mohajir
222	7	2(1)	35,42,43,50,54^b^,60,?	47,53^b^	Lung (60), prostate (70), lung	Kashmiri

*BC* breast cancer, *OC* ovarian cancer, *Unknown* cancer phenotype is not known

^a^Mutations previously described [11, 18]; ^b^Proband; ^c^Age at cancer diagnosis is mentioned along with cancer phenotype. For relatives with unknown age at cancer onset, only cancer phenotype is mentioned; ^d^Families with male breast cancer

**Fig. 2:**
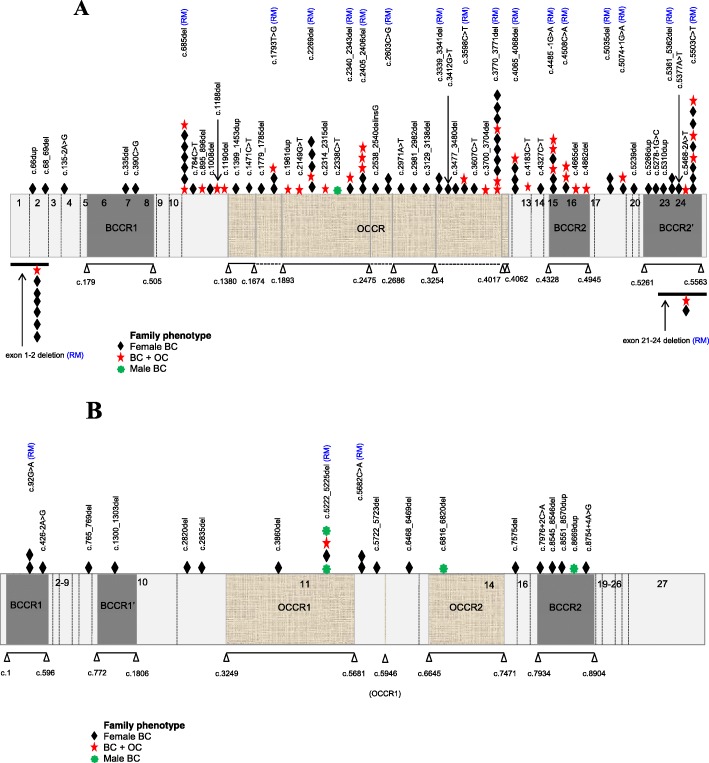
Distribution of deleterious germline mutations identified in Pakistani breast/ovarian cancer families across the *BRCA1* and *BRCA2* genes. Recurrent mutations are marked with (RM). The distribution of breast cancer (BC) and ovarian cancer (OC) in families according to the position of the mutations in *BRCA1* (**a**) and *BRCA2* (**b**) is shown. Regions inferred to be breast cancer cluster regions (BCCRs) and ovarian cancer cluster regions (OCCRs) according to Rebbeck and colleagues [24] are shown at the bottom

Twenty-one (21/71; 29.6%) mutations including 18 in *BRCA1* and three in *BRCA2* occurred more than once (Fig. 2a, b). These mutations were identified in 83 unrelated families and accounted for 62.4% (83/133) of all families with mutations. The most common *BRCA1* mutation was c.3770_3771del (ten Punjabi families), followed by c.5503C>T (nine Punjabi families), exon 1-2 deletion (seven Punjabi families), c.685del (five Punjabi and two Balochi families), c.4485-1G>A (six Punjabi families), c.2269del (one Punjabi and four Pathan families), c.2405_2406del (five Punjabi families), c.4065_4068del (three Punjabi and one Pathan families), c.1793T>G and c.4508C>A (three Punjabi families each), exon 21-24 deletion, c.2603C>G, c.3339_3341del, c.3598C>T, c.5035del, c.5074+1G>A, and c.5361_5362del (two Punjabi families each) and c.2340_2343del (one Pathan and one Mohajir families). The most common *BRCA2* mutation was c.5222_5225del (one Punjabi and three Mohajir families), followed by c.92G>A (two Punjabi families) and c.5682C>A (two Pathan families).

In addition to the deleterious mutations, 153 (28.4%) distinct *BRCA1/2* sequence variants were detected: 79 missense variants, 48 non-coding variants, 24 synonymous variants, one in-frame deletion, and one polymorphic nonsense variant in exon 27 of *BRCA2* (data not shown).

### *BRCA1/2* mutation frequencies

The frequencies of *BRCA1/2* mutations by risk group are provided in Table 1. For *BRCA1,* the highest mutation frequency was noted in families with breast and ovarian cancer (53.8%), followed by families with at least three breast cancer cases (24.8%), families with two breast cancer cases (18.3%), or families with one early-onset breast cancer case (<30 years) (10.8%). For *BRCA2*, the highest frequency was observed in families with male breast cancer (14.8%).

### Patient and tumors characteristics by *BRCA1/2* status

*BRCA1* carriers (n=110) were more often identified among female patients (99.1% *vs.* 94.6%, *p*=0.039) and belonged to the Punjabi ethnic group (81.8% *vs.* 68.7%, *p*=0.030) compared to non-carriers (n=406). In contrast, *BRCA2* carriers (n=23) were more common among male patients (17.4% *vs.* 5.4%, *p*=0.043) and more often belonged to Pathan ethnic group (34.8% *vs.* 15.5%, *p*=0.009).

Female breast cancer patients with mutations in *BRCA1* (n=106) or *BRCA2* (n=19) had a similar mean age of diagnosis (34.0 years (range 21–72) and 37.7 years (range 23-56), respectively, *p*=0.073, Wilcoxon rank-sum test), which did not differ to that of non-carriers (n=377) (35.7 years (range 18-78). In contrast, male breast cancer patients harboring *BRCA2* mutations (n=4) had an older mean age of diagnosis than non-carriers (n=22) (66.5 years (range 54-76) and 52.5 years (range 27-69) years, respectively, *p*=0.039, Wilcoxon rank-sum test).

*BRCA1-*associated breast tumors more often were invasive ductal carcinomas (99.0% *vs.* 91.4%, *p*=0.004), triple-negative (60.8% *vs.* 22.6%, *p*=<0.0001), and of higher tumor grade (grade 3: 94.9% *vs.* 63.2%, *p*=<0.0001) compared to tumors of non-carriers. *BRCA2-*associated breast tumors more often were PR positive compared to tumors of non-carriers (81.8% *vs.* 57.2%, *p*=0.025) (data not shown).

## Discussion

To our knowledge, this is the largest Pakistani study that comprehensively investigated the spectrum of *BRCA1/2* small-range mutations and LGRs and prevalence of mutations in 539 high-risk families. Mutations were identified in 24.7% (133/539) of families. Eighteen *BRCA1* and three *BRCA2* mutations were recurrent and accounted for 68.2% and 34.8% of all mutations in *BRCA1* and *BRCA2*, respectively. Nine mutations were specific to the Pakistani population, whereas other mutations had been reported elsewhere.

The most common type of identified mutations were frameshift mutations (60.6%) followed by nonsense mutations (25.4%). These data are consistent with our previous report [10] and a recent worldwide study [25]. In Pakistani patients, *BRCA1* mutations were about 5-times more frequent than *BRCA2* mutations. A similar distribution was observed in two Asian studies from South India [26] and Saudi Arabia [27] and most studies among white populations [3–5, 28]. This is likely due to the predominance of recurrent *BRCA1* mutations in these populations. Contradictory results were reported in other Asian studies from China, Hong Kong, Korea, and Indonesia, where *BRCA2* mutations were observed at an equal or a higher frequency than *BRCA1* mutations [6, 12, 15–17].

Among the 133 mutations identified in our study, 18 *BRCA1* and three *BRCA2* mutations were recurrent, accounting for 68.2% and 34.8% of all mutations in *BRCA1* and *BRCA2*, respectively. The proportion of recurrent *BRCA1* mutations to the total number of identified *BRCA1* mutations is higher than our previous report [10], which is likely due to the larger size of the present study. Of the identified recurrent mutations, the majority was also reported as recurrent mutations in other populations [1, 4, 25], while few were exclusively identified in a specific ethnic group of Pakistan. Fourteen *BRCA1* mutations (c.3770_3771del, c.5503C>T, c.4485-1G>A, c.2405_2406del, c.1793T>G, c.4508C>A, c.2603C>G, c.3339_3341del, c.3598C>T, c.5035del, c.5074+1G>A, c.5361_5362del, exon 1-2 deletion, and exon 21-24 deletion) and one *BRCA2* mutation (c.92G>A) were identified only in the Punjabi ethnic group. One *BRCA2* mutation (c.5682C>A) was found only in the Pathan ethnic group. Five other recurrent mutations were identified in more than one ethnic group. Our findings imply that a panel of ethnic specific recurrent mutations may be useful for initial screening of high-risk patients from these ethnic groups. Founder effects were previously suggested for six of the 18 recurrent *BRCA1* mutations (c.3770_3771del, c.4065_4068del, c.4485-1G>A, c.4508C>A, c.5503C>T, exon 1-2 deletion) [9–11], while haplotype analyses of the remaining recurrent mutations have not been performed so far. The high percentage of recurrent *BRCA1* mutations facilitates the development of a local, economical, and efficient ethnic-specific genetic testing strategy in Pakistan.

*BRCA1/2* mutations were identified in 24.7% of Pakistani breast cancer families. This frequency is higher than that from our initial report (17%) [10], probably due to the larger study size and comprehensive mutation analyses of both genes. This frequency is also higher than those from other Asian reports from Hong Kong, Malaysia, and Korea, ranging from 9.4% to 21.7% [6–8, 16, 17]. These findings further support the notion that the *BRCA1/2* mutation frequencies vary among different populations. Our data are similar to those reported in white populations, ranging from 17.6% to 29.8% [1, 2, 4]. We found the highest mutation frequency in breast and ovarian cancer families (55.4%), in agreement with previous studies from Pakistan [10], Korea [16], and studies in white populations [4, 28]. We observed a 2.52 fold (53.8% *vs*. 21.3%) increased occurrence of *BRCA1* mutations in breast and ovarian cancer families compared to breast cancer only families, in line with previous reports [1, 4, 6, 28]. Our findings support the notion that the presence of ovarian cancer in Pakistani breast cancer families increases the likelihood for the occurrence of *BRCA1* mutation.

In the present study on 27 families with male breast cancer, a *BRCA1/2* mutation frequency of approximately 19% was observed, with *BRCA2* mutations being about 4-times more common than *BRCA1* mutations. Our data are in line with previous studies [4, 14]. This observed frequency is higher than that reported in our initial much smaller study, in which no mutations were identified [10]. In agreement with the National Comprehensive Cancer Network (NCCN) guidelines, our data also warrant *BRCA1/2* testing in families with male breast cancer (NCCN Guidelines Version 2.2019).

The main strength of this study is its large size of 539 high-risk families, the comprehensive screening of both genes for small-range mutations and LGRs using highly sensitive methods (allowing the identification of recurrent *BRCA1/2* mutations in the Pakistani population and the more accurate estimation of *BRCA1/2* mutation frequencies among high-risk families), and the confirmations of mutations in an independent patient’s sample. However, our study also has some limitations. Participants were recruited at one tertiary care cancer center in Lahore, which may have introduced selection bias. Families belonging to Punjabi and Pathan ethnic groups are over-represented and, therefore, mutations in these groups may be over-represented. Nevertheless, Punjabi (44.7%) and Pathan (15.4%) are the most common ethnic groups reported in Pakistan (The World Factbook). Further, our data are based on self-reported ethnicity of study participants, which may lead to a misclassification of the ethnic origin of some of them.

## Conclusions

In summary, our study showed that *BRCA1/2* mutations account for 24.7% of high-risk breast cancer patients in Pakistan. Our results have important clinical implications, such as personalized treatment with platinum-based or PARP-inhibitor therapy for breast/ovarian cancer patients carrying a pathogenic *BRCA1/2* mutation and early detection, surgical prevention, and chemoprevention strategies for their unaffected *BRCA1/2* mutation positive relatives. Overall, *BRCA1/2* mutations account for one in four patients with a family history of breast cancer/breast and ovarian cancer, one in five patients with male breast cancer, and one in eight patients with early-onset breast cancer. Eighteen mutations in *BRCA1* and three in *BRCA2* were recurrent and accounted for 68.2% and 34.8% of all identified mutations in *BRCA1* and *BRCA2*, respectively. Our data suggest that *BRCA1* testing may be justified for families with multiple female breast cancers, breast and ovarian cancer or early-onset breast cancer and *BRCA2* testing for families with male breast cancer from Pakistan. Our findings will help in tailoring cost-effective genetic testing approach for the high-risk Pakistani population or for individuals of Pakistani origin residing in other countries.

## Data Availability

The datasets used and/or analyzed during the current study are available from the corresponding author on reasonable request.

## Abbreviations

BIC: Breast cancer information core
HGVS: Human genome variation society
LGRs: Large genomic rearrangements
LOVD: The Leiden open variation database
SKMCH&RC: Shaukat Khanum Memorial Cancer Hospital and Research Centre
